# Effects of Social Media and Mobile Health Apps on Pregnancy Care: Meta-Analysis

**DOI:** 10.2196/11836

**Published:** 2019-01-30

**Authors:** Ko Ling Chan, Mengtong Chen

**Affiliations:** 1 Department of Applied Social Sciences The Hong Kong Polytechnic University Hong Kong China (Hong Kong)

**Keywords:** mHealth, social media, pregnancy, postpartum, maternal health

## Abstract

**Background:**

The use of social media and mobile health (mHealth) apps has been increasing in pregnancy care. However, the effectiveness of these interventions is still unclear.

**Objectives:**

We conducted a meta-analysis to examine the effectiveness of these interventions with regard to different health outcomes in pregnant and postpartum women and investigate the characteristics and components of interventions that may affect program effectiveness.

**Method:**

We performed a comprehensive literature search of major electronic databases and reference sections of related reviews and eligible studies. A random effects model was used to calculate the effect size.

**Results:**

Fifteen randomized controlled trial studies published in and before June 2018 that met the inclusion criteria were included in the meta-analysis. The interventions were effective in promoting maternal physical health including weight management, gestational diabetes mellitus control, and asthma control with a moderate to large effect size (*d*=0.72). Large effect sizes were also found for improving maternal mental health (*d*=0.84) and knowledge about pregnancy (*d*=0.80). Weight control interventions using wearable devices were more effective.

**Conclusion:**

Social media and mHealth apps have the potential to be widely used in improving maternal well-being. More large-scale clinical trials focusing on different health outcomes are suggested for future studies.

## Introduction

Every pregnancy is unique but carries risks of a number of physical and psychological problems. Low maternal well-being during pregnancy can negatively impact women’s health outcomes and child development [1]. For example, overweight and obesity have become a common health problem associated with pregnancy in both developed and developing countries with dramatically increased prevalence over the past two decades [2,3]. Overweight and obesity before, during, and after pregnancy increase the risk of diseases such as metabolic syndrome, cardiovascular disease, and diabetes, as well as a number of child developmental problems such as preterm birth, low birth weight, neurodevelopmental delay, and immune and infectious disease, further increasing medical costs and negatively influencing family well-being [3-5]. Approximately 7.5% of pregnant women suffer from gestational diabetes mellitus (GDM), and the prevalence is significantly higher among Asian and Pacific Islanders [6]. Pregnant women with GDM, in particular those having obesity and overweight problems, are at significantly higher risk of adverse pregnancy outcomes [7]. Depression among women during and after pregnancy can also have negative effects on maternal health and interpersonal functioning, which is a common and persistent mental health problem [8,9].

Effective interventions that can help reduce risks during pregnancy and improve maternal well-being therefore play an important role. Research has shown that in addition to regular check-ups, several other forms of pregnancy care provided by medical professionals, therapists, and social workers are useful means to improve maternal well-being during pregnancy, such as yoga and physical activity, lifestyle, mindfulness, and psychotherapeutic interventions [10-14]. However, these traditional health services are often restricted by time and place, as working parents may not be able to attend during the daytime. Women from disadvantaged groups often have limited resources, which prevent their access to health services [15,16]. In addition, these women were found to have poor treatment adherence and high attrition, which resulted in nonsignificant changes after the services [17]. From a service provider point of view, traditional services for pregnancy care often involve a number of health professionals providing face-to-face treatment, which is quite expensive and cannot reach different populations [18].

In recent years, mobile technologies have been widely used in the provision of pregnancy care services, benefiting from the rapid development of information communication technology (ICT) and universal access to these technologies [16]. More social media and mobile health (mHealth) apps are being used today, taking the place of traditional text message or email services. Social media websites provide women with a platform for obtaining health information and interactions with health professionals and peers [19]. Because of the increasing ownership rate of mobile phones, a large number of mHealth apps on health topics have been developed and are installed by consumers [18]. In addition to quick and easy access to health information, mHealth apps can improve interactions with the health care system—for example, consumers can monitor their health conditions by recording or uploading health status data using the apps [20]. Many apps can also promote health behaviors such as maintaining sufficient physical activity and having a healthy diet [21].

Pregnant and postpartum women are increasingly relying on social media and mHealth apps as sources of health information and services for self-care and infant care [22,23]. Systematic reviews show that the use of mHealth apps and social media is feasible and acceptable to support pregnancy care, including promoting a healthy lifestyle and providing health information in high-income countries [16,24]. However, the effectiveness of the interventions using mHealth apps and social media is still unclear, and the ways that diverse intervention components contribute to program effectiveness is also unclear.

We conducted a meta-analysis to examine the effectiveness of mHealth apps and social media interventions for pregnant and postpartum women by calculating the effect size and examining the characteristics of these interventions that may be related to program effectiveness.

## Methods

### Search Method

Study procedures followed Preferred Reporting Items for Systematic Reviews and Meta-Analyses (PRISMA) guidelines. We conducted a comprehensive literature search in online databases including PsycInfo, PsycARTICLES, Sociological Abstracts, Social Services Abstracts, Medline, ERIC, CINAHL Complete, and PubMed. We searched relevant studies published in and before June 2018. Advanced searches in titles, keywords, and abstracts were performed using the combinations of three groups of terms: (1) mobile technology and social media, including smartphone, mobile phone, social networking, Facebook, Twitter, WhatsApp, WeChat, and virtual reality; (2) pregnancy status, including pregnancy, pregnant, gestation, postnatal, and postpartum; (3) pregnancy care, including intervention, program, treatment, prevention, education, and therapy. In addition to the electronic database search, we hand-searched the reference lists of retrieved studies and relevant reviews, as well as grey literature including conference abstracts and dissertations.

### Inclusion and Exclusion Criteria

The literature search aimed at identifying original evaluation studies on mHealth apps and social media interventions for pregnancy care. Eligible studies should (1) focus on interventions providing pregnancy care, including prenatal and postpartum health care for expectant mothers; (2) use advanced technology, such as mobile apps for social media or health care; (3) aim at health outcomes such healthy pregnancy and maternal well-being; (4) use experimental or quasi-experimental design; (5) report enough data to calculate the effect size; and (6) be published in English or Chinese.

Studies were not included if they (1) examined the use of mobile technology among health workers, (2) used a qualitative evaluation method only, or (3) used a traditional method such as providing short message services (texts) or sending emails.

### Data Extraction

In the first step, we designed a standardized form to code study characteristics. The study publication information (author, contacts, publication year, and country), methodological characteristics (study design, sample size, and the use of clinical sample or community sample), intervention details (aim, content, device, mHealth or social media apps, duration, attrition rate, and service provider), and participant profiles (age, pregnancy status, health status, and socioeconomic status) were recorded using this form. In the second step, we coded study outcomes and extracted data (eg, mean, standard deviation, *P* values, sample sizes) for effect size calculation. The outcomes include health outcomes of pregnant or postpartum women such as pregnancy weight control, asthma control, health knowledge, and stress and depression management. Two authors performed data extraction separately, and disagreements were resolved by consensus.

### Quality Assessment

To obtain a valid estimate of intervention effectiveness and reduce the risk of bias in the meta-analysis, we used a checklist to assess the methodological quality of the included studies. The checklist (see Multimedia Appendix 1) is composed of eight items, measuring study design, participant eligibility criteria, sample size calculation, randomization process, intervention details, participant profiles, primary outcomes, and statistical methods. Each item is allocated 1 point; therefore, the highest score is 8 if all criteria are met, and scores of 5 and above are regarded as satisfactory. Two researchers in the research team evaluated the studies independently. To measure rater agreement, the Cohen kappa coefficient was used. The level of agreement was high between the two raters. Disagreements were resolved by discussion with the first author through a consensus-building process.

### Statistical Analysis

**Figure 1:**
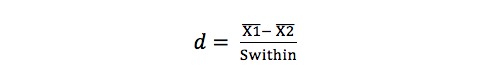
Formula for calculating the Cohen *d* statistic.

First, we provided a complete summary of the studies by tabulating publication information, methodology, intervention, and participant characteristics. Second, we calculated the effect size of each study using the Cohen *d* statistic. The Cohen *d* was calculated using the formula seen in Figure 1, in which the difference between two means is divided by a standard deviation for the two groups [25]. If a study reported multiple outcomes, the mean effect size of these outcomes was used. If there were studies based on the same intervention program, they were merged into one study and we calculated the mean effect size for these studies. The overall pooled effect size of the included studies was calculated based on a random effects model because of the different features of the interventions. The *Q* statistic and *I*^*2*^ were used to measure the variation in study outcomes between different studies. In addition, we used the *Q* statistic to test the effect of moderator variables, which may be related to program effectiveness. To examine whether publication bias occurred in our meta-analysis, we constructed a funnel plot. A symmetric inverted funnel plot indicates an absence of publication bias with high probability. Statistical analyses were performed using the Comprehensive Meta-Analysis (CMA) 3.0 program (Biostat Inc).

## Results

### Study Characteristics

#### Study Selection Process

**Figure 2:**
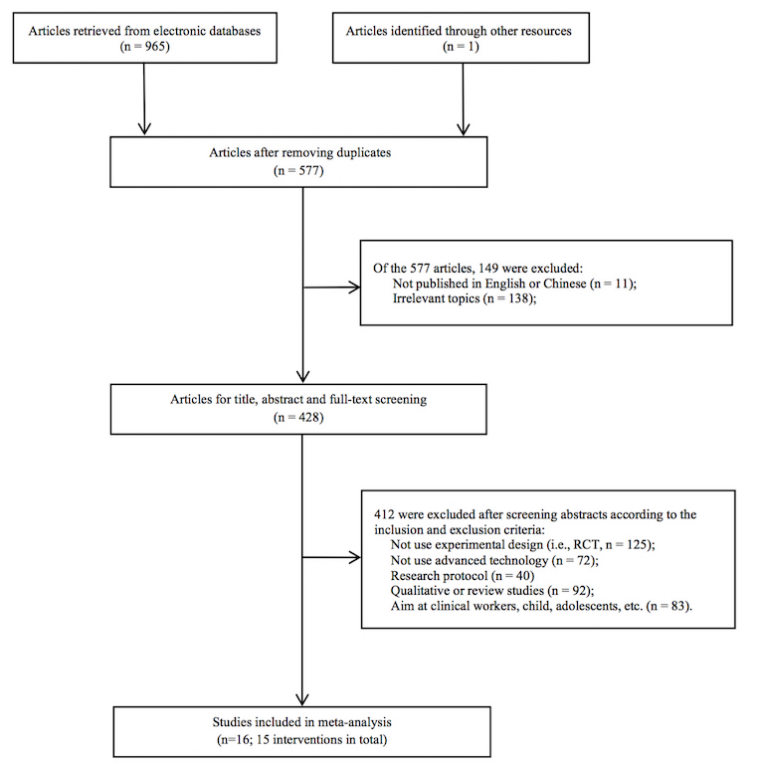
Flow diagram of study selection.

Figure 2 shows the results of the literature search and the study selection process. The literature search yielded 577 citations after removing duplicate records. A total of 149 articles were excluded because they were not published in English or Chinese or were focused on irrelevant topics. Then, on the basis of title, abstract, and full-text screening, 412 research articles were excluded based on the inclusion and exclusion criteria. Finally, 16 articles were found to be suitable for inclusion. Because two research articles [26,27] were based on one intervention program, a total of 15 studies were synthesized in the meta-analysis.

#### Methodological Characteristics

Multimedia Appendix 2 summarizes the publication information and methodological characteristics of each included study. All of the included studies were published in or after 2014, in line with the rapid development and spread of ICT in recent years. The studies were conducted in diverse countries and regions, including the United States, Australia, United Kingdom, Ireland, Israel, Indonesia, China, and Taiwan.

All 15 studies used randomized controlled trials (RCTs) to evaluate the effectiveness of these technology-based interventions. The sample size of the included studies ranged from 16 to 1689 participants, with a mean sample size of 225 and a median sample size of 87. All of the studies investigated a clinical sample of pregnant or postpartum women. With satisfactory methodological quality scores, all of the studies were graded at low risk of bias.

#### Intervention Components

Multimedia Appendix 3 summarizes the intervention characteristics and outcomes of each included study. Although all of the selected interventions aimed at improving maternal well-being, they involved different contents, and the approaches providing services differed to some extent.

Intervention in lifestyle was the major content in 14 selected studies. One approach was through psychoeducation. For example, pregnant and postpartum women obtained health information, identified risk behaviors and situations, learned to set achievable goals, and used behavior skills [28,29]. Thus, the participants were expected to manage their weight or control glucose by increasing their physical activity and changing dietary intake [27,30-32]. In addition, with knowledge about maternal and infant health, the participants increased their birth preparedness, complication readiness, and feeding behaviors [33,34]. Participants often accompanied the psychoeducation approach with self-monitoring to promote their lifestyle change, in which parents were required to maintain regular physical activities and pay attention to dietary intake. Patient monitoring devices such as handheld respiratory devices [35] and wearable devices such as Fitbit (Fitbit Inc) [30,31,36] were often used as support tools to track participants’ physical activities and record health status.

In addition to lifestyle intervention, there was one social support program developed by Cheng et al [37] delivered via mobile phone to reduce postpartum perceived stress and depression. Participants received emotional support as well as information about maternal and infant care from professionals via the social media instant communication app Line.

Ten interventions included a socially interactive component. Social media platforms (eg, Facebook, Line, and WeChat) not only provided a forum for knowledge sharing and behavior skills training, but also enabled participants easy access to support from clinical professionals and peer groups. Interactive components also increased peer support and promoted participant engagement [17,27]. Although clinical professionals were not necessarily involved in service providing because of the nature of mHealth, professional consultations by health coaches, psychologists, dietitians, physicians and obstetric doctors were still provided in interventions with interactive components.

Pregnancy care was either provided via social media platforms or via mobile phone platforms such as mHealth apps. A reminder function was used in apps to encourage participants to use the service and change their health behaviors. The interventions were generally long term, which covered most of the long gestation period and/or postpartum period.

#### Participant Profiles

With the exception of the study by Santoso et al [34], which included pregnant couples, all of the interventions were designed exclusively for women. The female participants were aged between 24 and 34 years with diverse socioeconomic characteristics and ethnicities. Participants who were African American or Hispanic and received Medicaid were particularly selected in the study by Herring et al [17,26,27]. Ten studies focused on overweight or obese women with a body mass index (BMI) above 25 kg/m^2^. Multimedia Appendix 4 summarizes the demographic characteristics of the sample.

### Intervention Effectiveness

**Figure 3:**
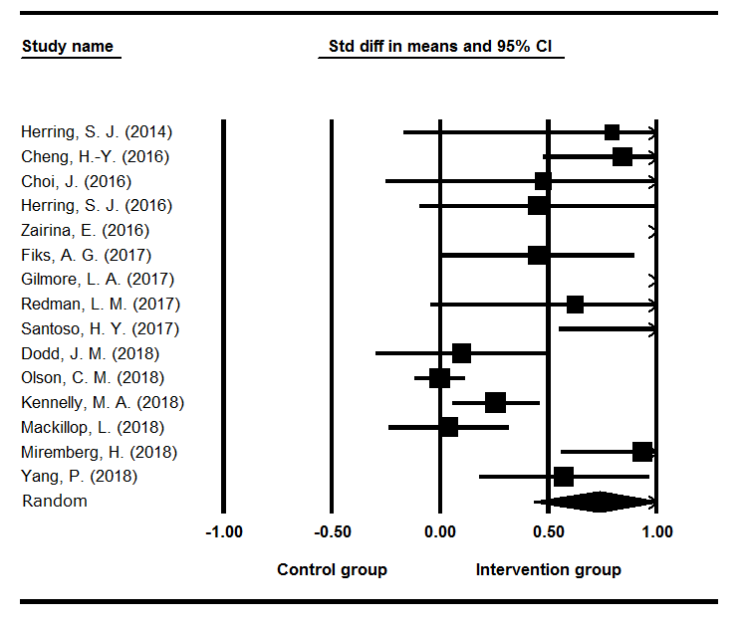
Effect size for each study.

Table 1 shows the effect size of each study pooled by different outcomes and time points. The results are displayed in a forest plot as shown in Figure 3. With the exception of the study by Olson et al [38], all of the interventions reported positive effects with an overall random effect size of 0.74 (*P*<.001, *Q*=146.45, *I*^*2*^=90.44). The overall effect size is considered medium to large, according to the Cohen criteria for effect size interpretation [39]. However, the large *Q* statistic indicated the wide variance in the effect sizes of different interventions, and it was estimated that 90.44% of the variance was due to heterogeneity. One reason for the large amount of heterogeneity is that the included studies aimed at different health outcomes. When we examined the effect sizes of different outcomes, the *Q* value decreased, showing that the variation is smaller for different specific outcomes.

As shown in Table 2, twelve studies aimed to improve the physical health outcomes of pregnant or postpartum women, including weight management (*d*=0.45, *P*=.003), GDM control (*d*=0.41, *P*=.03), and asthma control (*d*=3.43, *P*<.001), with an overall random effect size of 0.72 (*P*<.001, *Q*=127.3, *I*^*2*^=91.36). One study aimed to improve maternal mental health (eg, reducing postpartum stress and depression), and the effect size was 0.84 (*P*<.001). Two studies aimed to improve knowledge about birth preparedness and infant feeding, and the effect size was 0.8 (*P*=.04, *Q*=3.55, *I*^*2*^=71.82).

**Table 1:** Effect size for each study pooled by outcomes and time points.

Study name	Effect size	Standard error	Lower limit	Upper limit	*P* value
Herring, SJ (2014)	0.80	0.49	–0.17	1.75	.11
Cheng, HY (2016)	0.84	0.19	0.48	1.21	<.001
Choi, J (2016)	0.48	0.37	–0.25	1.20	.20
Herring, SJ (2016)	0.45	0.28	–0.09	1.00	.10
Zairina, E (2016)	3.43	0.38	2.69	4.17	<.001
Fiks, AG (2017)	0.45	0.23	0.003	0.90	.048
Gilmore, LA (2017)	2.05	0.43	1.21	2.89	<.001
Redman, LM (2017)	0.63	0.34	–0.05	1.30	.07
Santoso, HY (2017)	1.25	0.36	0.55	1.94	<.001
Dodd, JM (2018)	0.10	0.20	–0.29	0.49	.62
Olson, CM (2018)	–0.002	0.06	–0.12	0.11	.97
Kennelly, MA (2018)	0.26	0.10	0.06	0.46	.01
Mackillop, L (2018)	0.04	0.14	–0.24	0.31	.78
Miremberg, H (2018)	0.94	0.19	0.56	1.32	<.001
Yang, P (2018)	0.57	0.20	0.18	0.96	.004
Total	0.74	0.16	0.43	1.04	<.001

**Table 2:** Effect sizes of social media and mHealth apps for different health outcomes.

Outcome		*k*^b^	ES^a^ and 95% CI				Test of null		Test of heterogeneity		
			*d*^c^	*SE*	*LL*^d^	*UL*^e^	*Z*	*P* value	*Q*	*P* value	*I^2^*
**Physical health**		12	0.72	0.18	0.37	1.07	4.04	<.001	127.30	<.001	91.36
	Weight management	8	0.45	0.15	0.16	0.74	3.01	.003	36.85	<.001	81.00
	Gestational diabetes mellitus control	4	0.41	0.19	0.04	0.78	2.16	.03	17.38	<.001	82.74
	Asthma control	1	3.43	0.38	2.69	4.17	9.06	<.001	0	>.99	0
Stress and postnatal depression		1	0.84	0.19	0.47	1.21	4.53	<.001	0	>.99	0
Birth preparedness knowledge		2	0.80	0.40	0.03	1.57	2.03	.04	3.55	.06	71.82
Total		15	0.74	0.16	0.43	1.04	4.72	<.001	146.45	<.001	90.44

^a^ES: effect size

^b^*k*: number of studies.

^c^*d*: effect size.

^d^LL: lower limit.

^e^UL: upper limit.

### Subgroup Analyses

We investigated factors that may moderate the program effectiveness, including whether the intervention included interactive treatment content, use of professional consultation or not, the type of technology used, use of a wearable device or not, and whether the participants were overweight or obese. As shown in Table 3, the use of a wearable device to track physical activity in interventions aiming at weight management was a significant moderator (*Q*_*b*_=5.91, *P*=.02). Using such a device resulted in a larger pooled effect size (*d*=0.97). Sample size was also a significant moderator of the effect size (*Q*_*b*_=7.38, *P*=.007). Studies with smaller sample sizes resulted in a larger pooled effect size (*d*=1.13).

Moderator analyses in Table 3 show that interactive content and professional consultation were not significant moderators (*Q*_*b*_=1.5, *P*=.22). The effects of interventions providing interactive treatment content and involving professional consultations were not significantly better compared with the interventions without these components. The interventions using social networking (*d*=0.67) and health and fitness mobile phone apps (*d*=0.77) were also similarly effective as the result of the *Q* test was insignificant.

**Table 3:** Moderator variable analyses.

Moderator group		*k*^a^	*d*^b^	*LL*^c^	*UL*^d^	*Q_b_*^e^	*P* value
**Interactive content**							
	Yes	10	0.60	0.18	1.03	1.51	.22
	No	5	1.08	0.46	1.70		
**Professional consultation**							
	Yes	10	0.60	0.18	1.03	1.51	.22
	No	5	1.08	0.46	1.70		
**Technology**							
	Social networking	6	0.67	0.21	1.14	0.10	.75
	Health and fitness mobile phone app	9	0.77	0.39	1.15		
**Wearable device^f^**							
	Yes	3	0.97	0.45	1.49	5.91	.02
	No	5	0.24	–0.05	0.52		
**Sample size**							
	Above 100	7	0.38	0.02	0.74	7.38	.007
	Below 100	8	1.13	0.73	1.54		

^a^*k*: number of studies.

^b^*d*: effect size.

^c^LL: lower limit.

^d^UL: upper limit.

^e^*Q*_*b*_: between-group heterogeneity

^f^Moderator effect in weight management.

### Publication Bias Analysis

**Figure 4:**
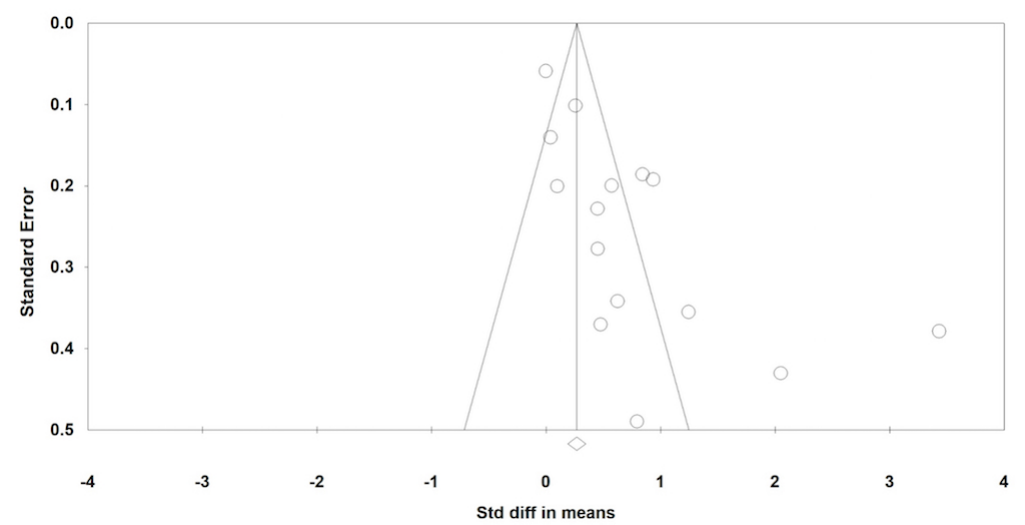
Funnel plot for publication bias.

A funnel plot was used to examine publication bias of our meta-analysis, as shown in Figure 4. Almost half of the studies used a larger sample (n>100), and they concentrated around the top of the funnel plot. However, there were more studies on the right side of the mean effect size, especially studies with smaller samples, which made the funnel plot asymmetric. This means that positive results of the interventions based on small sample sizes were more likely to be published. Therefore, there is evidence that publication bias exists.

## Discussion

### Principal Findings

The use of social media and mHealth apps has been increasing in pregnancy care because of the low cost and their easy access regardless of time and geographic location [40]. This meta-analysis synthesized the findings of 15 RCT studies conducted in different countries and regions and provides evidence on the effectiveness of these technology-based interventions in providing health care services to pregnant and postpartum women. Moderate to large effect sizes were found in regard to different health outcomes including maternal physical health, mental health, and knowledge about pregnancy. In addition, we investigated the characteristics and components of the interventions that may affect program effectiveness.

### Effects on Maternal Physical Health

Pregnancy is a life-changing experience with risks of excessive weight gain and obesity [36]. Postpartum weight retention is a prevalent problem among US women, especially within racial and ethnic minorities [17]. However, it can be difficult for pregnant or postpartum women to manage their weight by increasing physical activity or changing dietary intake because of limited resources in health care or a low level of engagement in health management [16,41]. This meta-analysis finds that there was a moderate effect in maternal weight control and maintaining optimal body composition by promoting lifestyle change and self-monitoring via mHealth apps and social media.

Similarly to pregnancy weight control, GDM control also relies on self-monitoring the change of unhealthy lifestyle and listening to clinical decisions, and patient compliance is particularly important [42,43]. The results of the meta-analysis show that mHealth apps and social media were also effective for pregnant women with GDM, with a small to moderate effect size. As participants in the intervention group only had half of the clinic visits compared with participants in the standard care group in the research of Mackillop et al [42], we estimate that the intervention can be more effective with the same number of clinical visits.

An mHealth app was found very effective in asthma control during pregnancy [35]. However, this study is just an initial step toward understanding the effect of the social media and mHealth apps in physical health outcomes other than weight or GDM control. The result of a case-control study showed that the mHealth app can be effective in urinary incontinence management during pregnancy [44]. We can estimate that mHealth apps can be applied in other health services. To conclude, the positive and significant effects demonstrate that lifestyle intervention using advanced technology can be effective in improving maternal health.

### Effects on Maternal Mental Health and Birth Preparedness

Psychological interventions delivered via mobile phones have been found effective in reducing depression and anxiety in existing meta-analyses [45,46]. However, whether the interventions are effective among pregnant or postpartum women is unclear. This study provided additional evidence that mHealth apps and social media can be useful in reducing pregnancy-related stress and depression. However, there was only one RCT study examining the effectiveness in maternal mental health outcomes [37].

Social media and mobile phone apps are becoming increasingly popular among pregnant women and their partners to access health knowledge and learn to identify risk behaviors and danger signs during pregnancy. The findings from two RCT studies [33,34] demonstrate the usefulness of the intervention programs to prepare the participants to become mothers. A pretest-posttest study found that providing information about maternal and infant care via the mHealth app can reduce maternal stress during pregnancy [47]. Therefore, with the improvement in health knowledge, maternal mental health may also improve.

### Factors Related to Intervention Effectiveness

Finally, we were interested in investigating whether intervention characteristics or components can affect their effectiveness. Interventions using social media and interventions using mobile phone apps resulted in similar effect sizes. In addition, it seems to make little difference whether interactive treatment content or professional consultation was provided in the intervention. One explanation may be that all apps have a reminder function and provide health information, which is similar to some functions of the interactive treatment and professional consultation. Another possible explanation may be that the usefulness of the interventions is more likely to rely on women’s self-monitoring. Therefore, different forms of mHealth apps and social media providing pregnancy care may have similar benefits. Interventions without interactive content or professional consultation can be more cost effective.

The results of moderator analyses also showed that using wearable devices to track participants’ physical activities has the potential to enhance program effectiveness in weight control during the prenatal or postnatal periods. The use of wearable devices may be a good way to improve self-monitoring. Another moderator variable that significantly contributed to the variance in the effect size was sample size. Interventions with smaller sample sizes seem to be more effective, whereas interventions with larger samples were less effective. Olson et al [38] and Dodd et al [28] argued that in their study, the similar contents of intervention and control groups or the low use of self-monitoring tools in the intervention may explain the low program effectiveness. However, the absence of small studies with small effect sizes also indicates publication bias among the studies in this meta-analysis.

### Strengths and Limitations

Because there was a lack of quantitative integration of the evidence on effectiveness of the social media and mHealth apps, further investigation was recommended before the implementation of the intervention [24]. This study includes rigorous studies that offer high-quality evidence. Our review is the first meta-analysis evaluating program effectiveness through credible statistical analyses.

There are several limitations to this study. First, the presence of publication bias indicates that more studies need to be included, as the studies included in the meta-analysis were more likely to report larger effect sizes. Second, as most studies included provided only limited details about participant profiles, several factors cannot be examined through moderator analysis such as participant socioeconomic status and health status.

### Implications for Research

Although the use of advanced technology in pregnancy care has been increasing in recent years and there are promising results in improving maternal health outcomes, research in this area is still in its early stages. First, more large-scale clinical trials are suggested in future studies. This is because interventions with smaller sample sizes are more likely to report larger effect sizes, which can result in skewed distribution of effect sizes in the meta-analysis. Also, more studies are suggested to be included in future reviews. Second, because interventions included in this review were used predominantly for managing health problems, the effectiveness in improving mental health of pregnant and postpartum women needs to be examined. Third, cost effectiveness could be an important feature of the use of mHealth apps and social media in pregnancy care [42]; however, it was not examined in most studies. Therefore, cost analysis is necessary in future studies.

### Implications for Practice

This meta-analysis of the effectiveness of social media and mHealth apps has several implications for future practice. First, interventions with the use of social media and mHealth apps can be effective in promoting maternal well-being. The positive effects in developing countries such as Indonesia and China imply that the use of mobile technologies in pregnancy care can be less restricted by social and economic development. Social media and mHealth apps can be widely adopted in different areas and have greater public health impact.

Second, the study of Santoso et al [34] demonstrated that fathers can also be positively involved in pregnancy care and birth preparedness by using social media and health apps. The inclusion of fathers could improve health outcomes for the whole family [1]. Future practice should consider attracting fathers to use the related services.

Third, the use of mHealth apps was poor among participants in some interventions, which may lead to low effectiveness [28,38]. Therefore, it is important for researchers, service providers, and app developers to consider how to increase the use of interventions and customer stickiness. It is also necessary to find useful ways to improve participant self-monitoring.

Fourth, even though this review included a number of mHealth apps, most commonly used mHealth apps are commercial and the credibility of their information is unknown [23]. Therefore, it is necessary to examine the quality and effectiveness of their services. Evidence-based mHealth apps and social media interventions for pregnant women are recommended in the practice.

### Conclusion

Social media and mHealth apps are increasingly used in pregnancy care with emerging promising findings. In this meta-analysis, we found the interventions were useful with moderate to large effect sizes in regard to maternal health, mental health, and knowledge about pregnancy. We conclude that social media and mHealth apps have the potential to be widely used in improving maternal well-being during the prenatal and postnatal periods. More large-scale clinical trials with comprehensive aims are suggested for future studies.

## Appendix

Multimedia Appendix 1 Methodological quality assessment checklist.

Multimedia Appendix 2 Publication information and methodological characteristics.

Multimedia Appendix 3 Intervention characteristics and study outcomes.

Multimedia Appendix 4 Characteristics of female participants.

## Abbreviations

BMI: body mass index
GDM: gestational diabetes mellitus
ICT: information communication technology
mHealth: mobile health
PRISMA: Preferred Reporting Items for Systematic Reviews and Meta-Analyses
RCT: randomized controlled trial
