# Supplementary material for: Effects of Social Media and Mobile Health Apps on Pregnancy Care: Meta-Analysis
Source: JMIR Mhealth Uhealth. 2019 Jan 30;7(1):e11836. doi: 10.2196/11836 (PMC6372934; doi:10.2196/11836)
Supplement: Multimedia Appendix 1 [file mhealth_v7i1e11836_app1.pdf]

Multimedia Appendix 1

Methodological Quality Assessment Checklist.

1. Does the study clearly describe the trial design? (1 point)

2. Are there eligibility criteria for participants? (1 point)

3. Is the sample size suitable? Sample size calculation has been performed. (1 point)

4. Does the study provide sufficient details of intervention program to allow replication? (1 point)

5. Is the randomization process described? (1 point)

6. Does the study provide sufficient information about participant profiles (1 point)

7. Are the study outcome measures completely defined? (1 point)

8. Do the statistical methods compare the outcomes of control group and intervention group? (1 point)
