# Supplementary material for: Effects of Social Media and Mobile Health Apps on Pregnancy Care: Meta-Analysis
Source: JMIR Mhealth Uhealth. 2019 Jan 30;7(1):e11836. doi: 10.2196/11836 (PMC6372934; doi:10.2196/11836)
Supplement: Multimedia Appendix 2 [file mhealth_v7i1e11836_app2.pdf]

Multimedia Appendix 2

Publication Information and Methodological Characteristics.

Author, year	Country/ Region	Sample size (IG/CG)	Method score	Attrition rate (%)	Comparison intervention
Herring et al, 2014 [17]	US	18 (9/9)	7	5.6	Standard care
Cheng et al, 2016 [37]	Taiwan	126 (61/65)	7	10	–
Choi et al, 2016 [30]	US	30 (15/15)	7	3.3	Fitbit
Herring et al, 2016 [27] & Herring et al, 2017 [26]	US	66 (33/33)	7	15.2	Standard care
Zairina et al, 2017 [35]	Australia	72 (36/36)	8	4.2	Regular antenatal visit
Fiks et al, 2017 [33]	US	87 (43/44)	7	18.4	Text message reminder
Gilmore et al, 2017 [31]	US	40 (20/20)	6	12.5	Standard care
Redman et al, 2017 [36]	US	36 (17/19)	7	–	Obstetrician usual care
Santoso et al, 2017 [34]	Indonesia	38 (19/19)	7	0	Counseling
Dodd et al, 2018 [28]	Australia	162 (77/85)	8	38.3	All components except for APP
Olson et al, 2018 [38]	US	1,689 (1,126/563)	8	11.7	Usual care
Kennelly et al, 2018 [41]	Ireland	565 (278/287)	8	11.9	Standard care
Mackillop et al, 2018 [42]	UK	206 (103/103)	8	1.5	Higher dose of standard care
Miremberg et al, 2018 [43]	Israel	126 (61/65)	8	5	Standard care
Yang et al, 2018 [32]	China	107 (57/50)	5	0	Standard care

Note. CG = control group, IG = intervention group
