# Supplementary material for: Effects of Social Media and Mobile Health Apps on Pregnancy Care: Meta-Analysis
Source: JMIR Mhealth Uhealth. 2019 Jan 30;7(1):e11836. doi: 10.2196/11836 (PMC6372934; doi:10.2196/11836)
Supplement: Multimedia Appendix 3 [file mhealth_v7i1e11836_app3.pdf]

Multimedia Appendix 3

Intervention Characteristics and Study Outcomes.

Author, year	Interactive	Consultation	Type	Devices	Overweight or obese	Outcome
Herring et al, 2014 [17]	Yes	Yes	Social media-Facebook	–	Yes	WM
Cheng et al, 2016 [37]	Yes	Yes	Social media-Line	–	–	MH
Choi et al, 2016 [30]	No	No	Health app	–	–	WM
Herring et al, 2016 [27] & Herring et al, 2017 [26]	Yes	Yes	Social media-Facebook	–	Yes	WM
Zairina et al, 2017 [35]	No	No	Health app	Handheld respiratory device	Yes	Asthma
Fiks et al, 2017 [33]	Yes	Yes	Social media-Facebook	–	Yes	BP
Gilmore et al, 2017 [31]	Yes	Yes	Health app	Fitbit	Yes	WM
Redman et al, 2017 [36]	No	No	Health app	Fitbit	–	WM
Santoso et al, 2017 [34]	No	No	Health app	–	–	BP
Dodd et al, 2018 [28]	Yes	Yes	Health app	Fitbit	Yes	WM
Olson et al, 2018 [38]	No	No	Website and mobile phone platform	–	No	WM
Kennelly et al, 2018 [41]	Yes	Yes	Health app	–	Yes	WM; GDMC
Mackillop et al, 2018 [42]	Yes	Yes	Health app	–	Yes	GDMC
Miremberg et al, 2018 [43]	Yes	Yes	Social media-communication platform	–	Yes	GDMC
Yang et al, 2018 [32]	Yes	Yes	Social media-WeChat	–	–	GDMC

Note. BP = birth preparedness; CG = control group; GDMC = gestational diabetes Mellitus control; IG = intervention group; MH = mental health; WM = weight management
