# Supplementary material for: Effects of Social Media and Mobile Health Apps on Pregnancy Care: Meta-Analysis
Source: JMIR Mhealth Uhealth. 2019 Jan 30;7(1):e11836. doi: 10.2196/11836 (PMC6372934; doi:10.2196/11836)
Supplement: Multimedia Appendix 4 [file mhealth_v7i1e11836_app4.pdf]

Multimedia Appendix 4

Characteristics of female participants

Author, year	Mean age (SD)	Education	Employment	Ethnicity	BMI > 25
Herring et al, 2014 [17]	24.2 (5.1)	66.7% high school or above	NA	78% black, 22% Hispanic	Yes
Cheng et al, 2016 [37]	33.13 (4.56)	65.9% college degree	73% employed	Chinese	NA
Choi et al, 2016 [30]	33.7 (2.6)	80% college degree	83.3% employed	40% Asian, 6.7% Black, 10% Hispanic/Latina, 43.3% White	NA
Herring et al, 2016 [27] & Herring et al, 2017 [26]	IG: 25.9 (4.9); CG: 25 (5.7)	16.7% some college or more	47% employed	African American	Yes
Zairina et al, 2017 [35]	31.4 (4.5)	62.5% college or more	48.6% employed	83.3% Caucasian, 8.3% Asian, 8.3% others	Yes
Fiks et al, 2017 [33]	26.5 (5.4)	36% college or above	45% employed	2% Hispanic/Latina, 88% black/African American, 6% white, 7% others	Yes
Gilmore et al, 2017 [31]	IG: 26 (5.2); CG: 27.2 (6.1)	31.3% college or above	NA	22.9% white, 74.3% black, 2.9% Asian	Yes
Redman et al, 2017 [36]	IG: 29 (4.2); CG: 29.5 (5.1)	63.9% college or above	NA	22.2% black, 75% white, 2.8% others	NA
Santoso et al, 2017 [34]	28.9	65% secondary level of education	66% employed	Indonesian	NA
Dodd et al, 2018 [28]	30.94 (5.65)	NA	NA	72.84% Caucasian	Half
Olson et al, 2018 [38]	NA	62.1% some college or above	NA	64% non-Hispanic white, 21.1% non-Hispanic African American, 6% Hispanic/Latina	Half
Kennelly et al, 2018 [41]	IG: 32.8 (4.6); CG: 32.1 (4.2)	84.6% some college or above	NA	91.2% Caucasian	Yes
Mackillop et al, 2018 [42]	IG: 33.9 (5.5); CG: 33 (5.6)	48.5% college or above	NA	77.7% White, 11.3% South Asian, 5% African/Caribbean, 2% East Asian, 4% others	Yes
Miremberg et al, 2018 [43]	IG: 31.7 (4.2); CG: 32 (6.3)	31% college or above	NA	NA	Yes
Yang et al, 2018 [32]	IG: 31.6 (4.2); CG: 32.2 (4.7)	NA	NA	Chinese	NA

Note. CG = control group, IG = intervention group
